# Gestational diabetes triggers postpartum cardiac hypertrophy via activation of calcineurin/NFAT signaling

**DOI:** 10.1038/s41598-021-00422-3

**Published:** 2021-10-22

**Authors:** Nirmal Verma, Sarah Srodulski, Sathya Velmurugan, Amanda Hoskins, Vivek K. Pandey, Florin Despa, Sanda Despa

**Affiliations:** Department of Pharmacology and Nutritional Sciences, University of Kentucky, 900 S Limestone, CTW 465, Lexington, KY 40536 USA

**Keywords:** Physiology, Cardiovascular biology

## Abstract

Population-based studies identified an association between a prior pregnancy complicated by gestational diabetes mellitus (GDM) and cardiac hypertrophy and dysfunction later in life. It is however unclear whether GDM initiates this phenotype and what are the underlying mechanisms. We addressed these questions by using female rats that express human amylin (HIP rats) as a GDM model and their wild-type (WT) littermates as the normal pregnancy model. Pregnant and two months postpartum HIP females had increased left-ventricular mass and wall thickness compared to non-pregnant HIP females, which indicates the presence of concentric hypertrophy. These parameters were unchanged in WT females during both pregnancy and postpartum periods. Hypertrophic Ca^2+^-dependent calcineurin/NFAT signaling was stimulated two months after giving birth in HIP females but not in the WT. In contrast, the CaMKII/HDAC hypertrophy pathway was active immediately after giving birth and returned to the baseline by two months postpartum in both WT and HIP females. Myocytes from two months postpartum HIP females exhibited slower Ca^2+^ transient relaxation and higher diastolic Ca^2+^ levels, which may explain calcineurin activation. No such effects occurred in the WT. These results suggest that a GDM-complicated pregnancy accelerates the development of pathological cardiac remodeling likely through activation of calcineurin/NFAT signaling.

## Introduction

Gestational diabetes mellitus (GDM) complicates between 6 and 26% of pregnancies worldwide^1–3^. Its incidence is growing rapidly, consistent with increasing rates of metabolic syndrome and obesity in the general population^1,4^ and more advanced maternal age^1^. While the condition is by definition transient, multiple studies found that women with a GDM-complicated pregnancy are at higher risk of developing type-2 diabetes^1,5–7^ and cardiovascular disease later in life^7–13^. In a cohort of > 47,000 women (10.3% were diagnosed with GDM), women with prior GDM had higher rates of non-invasive cardiac diagnostic procedures, angina pectoris and cardiovascular hospitalizations over a 10 year follow up period compared to women with uncomplicated pregnancies^10^. Another large (> 1,500,000 pregnancies) study^8^ revealed an association between GDM and higher risk of angina, myocardial infarction and hypertension within 7 years postpartum. The association was maintained after adjusting for age, diabetes, obesity and hypertensive disorders in pregnancy. In yet another cohort that included over 1,000,000 women, GDM was associated with a higher cumulative incidence of hospitalization for cardiovascular disease 25 years after delivery^11^. A meta-analysis of data from over 5 million women revealed that GDM remains strongly associated with risk of future cardiovascular events even in women that do not develop type-2 diabetes^13^.

Further insights into the link between GDM and the risk for cardiovascular disease were provided by studies that compared heart structure and function in women with GDM versus women with normal pregnancies. Prospective studies reported larger left ventricular relative wall thickness^14,15^ and mass^15^ in women with GDM compared to those in women with normal pregnancies at 34–39 weeks of gestation. Appiah et al.^12^ analyzed data of all women from the Coronary Artery Risk Development in Young Adults (CARDIA) study that had echocardiograms recorded at the beginning and at the end of a 20 years period and went through at least one pregnancy in between (609 women in total, 64 with GDM). After adjusting for potential confounders including body mass index, blood pressure, lipid levels and incident type-2 diabetes, women with prior GDM had increased left-ventricular mass index, impaired relaxation and systolic dysfunction at the end of the study. Notably, human studies cannot distinguish between GDM playing a causal role in this cardiac remodeling or being just an early, separate marker of a higher cardio-metabolic risk in particular individuals.

On short term, cardiac hypertrophy is a physiological, adaptive response to stress. Long term however, hypertrophy becomes pathological and is associated with a cellular switch to fetal gene expression, diastolic and systolic dysfunction and the occurrence of arrhythmias. Ultimately, pathological hypertrophy can progress to heart failure. Physiological cardiac hypertrophy occurs in normal pregnancy^16–19^ as a result of volume overload (caused by the large increase in blood volume) and endocrine changes, particularly the surge in progesterone and estrogen^18–20^. The molecular mechanisms underlying heart growth in pregnancy include activation of PI3K/Akt and MAPK/ERK pathways^18,21^. In humans, heart size returns to the pre-pregnancy level within a few months after giving birth^16,17^. Meanwhile, pathological hypertrophy is a major manifestation of heart disease in individuals with type-2 diabetes^22–27^ and occurs even in the absence of vascular complications^26,27^.

Studies in animal models revealed that Ca^2+^-dependent hypertrophy signaling, usually associated with pathological hypertrophy, contributes to heart growth in both type-2 diabetes^28^ and normal pregnancy^18,29^. Both CaMKII/HDAC and calcineurin/NFAT signaling pathways are activated in rats with late-onset type-2 diabetes^28^. Notably, the signaling was initiated in the prediabetic, insulin-resistant phase, before the onset of overt hyperglycemia^28^. In mice, the level and activity of calcineurin were elevated early in pregnancy and decreased below the baseline level in late pregnancy^18,29^. Moreover, inhibition of calcineurin by cyclosporine A blocked pregnancy-induced hypertrophy in mice^29^, which indicates that the calcineurin/NFAT pathway is not a simple bystander but actively contributes to cardiac hypertrophy in normal pregnancy.

While the mechanisms underlying cardiac hypertrophy during and after a GDM-complicated pregnancy are unknown, it is conceivable that some mechanisms are shared with normal pregnancy and type-2 diabetes. Here we used female rats with β-cell specific expression of the human isoform of the pancreatic hormone amylin and their wild-type littermates to assess the role of Ca^2+^-dependent hypertrophy signaling in the cardiac remodeling triggered by GDM. We found that two months after a GDM-complicated pregnancy, female rats showed cardiac hypertrophy and activation of calcineurin/NFAT signaling. In contrast, the CaMKII/HDAC hypertrophy pathway was active at term and returned to baseline after giving birth in rats with both normal and GDM-complicated pregnancies. These data suggest that calcineurin/NFAT pathway may play a role in the cardiac hypertrophy that occurs in females with a prior GDM pregnancy.

## Results

### Gestational diabetes in HIP rat females

**Figure 1:**
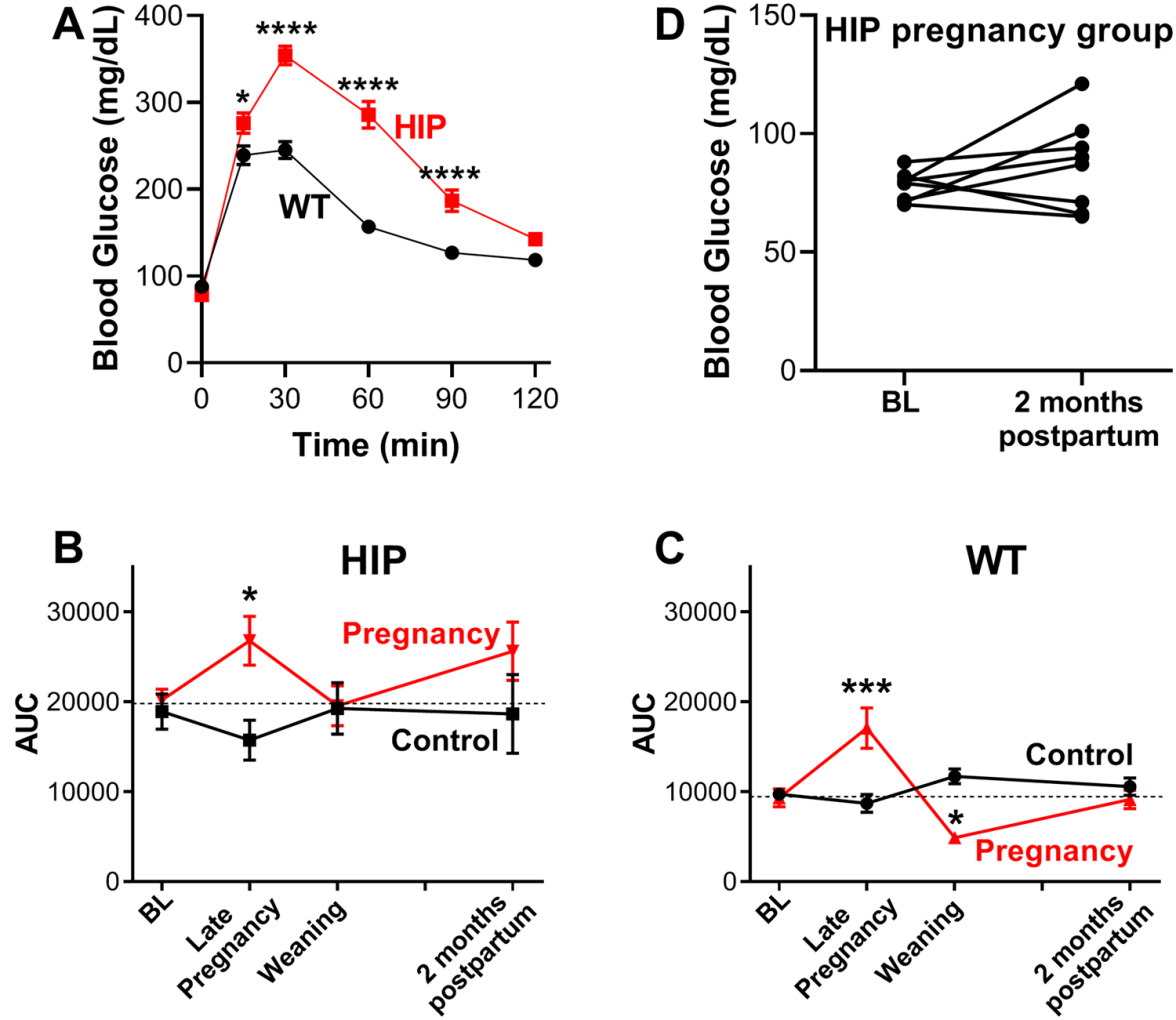
HIP females as a model of gestational diabetes. (**A**) HIP females (5–6 months of age) have reduced glucose tolerance compared to WT littermates, but fasting blood glucose is similar (n = 25 for WT, 30 for HIP). (**B,C**) Area under the curve in glucose tolerance tests performed in HIP (**B**) and WT (**C**) females in the pregnancy (N = 17 HIP and 14 WT in total) and control (N = 13 HIP and 11 WT in total) groups at baseline (BL), late (19–20 days) pregnancy, at the time of weaning the pups and 2 months postpartum. (**D**) Fasting blood glucose in HIP females from the pregnancy group at baseline and 2 months postpartum (N = 8). Statistical analysis was performed using Two-way ANOVA with Bonferroni’s multiple comparisons post-test for data in panels A-C and paired Student’s t-test for data in panel D.

HIP rats^28,30,31^ are obese Sprague–Dawley rats with pancreatic β-cell specific expression of the human variant of amylin, a pancreatic hormone that is processed in the same secretory vesicles as insulin and co-secreted with it^32^. At 5–6 months of age, amylin level is ~ fourfold higher in plasma from heterozygous HIP versus WT females (Supplementary Fig. 1A). Amylin acts as a satiation agent by activating receptors in the brain and slows down the gastric fluxes^33,34^. In agreement with this function, HIP females had lower body weight compared to their WT littermates (Supplementary Fig. 1B). Moreover, amylin was previously shown to reduce insulin-stimulated glucose uptake in muscle^35,36^. Indeed, HIP females (5–6 months of age) showed impaired glucose tolerance compared to age-matched WT females (Fig. 1A) while fasting blood glucose level was similar (see time 0 in Fig. 1A). To avoid confounding factors introduced by these differences between WT and HIP females at baseline, we designed the study to compare longitudinally females that went through pregnancy with age- and genotype-matched females that were never pregnant.

At this stage, HIP and WT females were randomly assigned to pregnancy and control study groups and further randomized for euthanasia and heart collection at 1 day or 2 months postpartum. Glucose tolerance decreased during pregnancy in both WT and HIP females, with HIP females remaining significantly glucose intolerant compared to the WT (Supplementary Fig. 2, *top*: time course of blood glucose level in glucose tolerance tests; Fig. 1B,C: area under the curve in these glucose tolerance tests). By the time of weaning, glucose tolerance returned to the pre-pregnancy level in HIP females (Supplementary Fig. 2, *middle*; Fig. 1B) and even improved compared to the control group in WT females (Supplementary Fig. 2, *middle*; Fig. 1C). In HIP females, glucose tolerance tended to worsen again by two months postpartum (Supplementary Fig. 2, *bottom*; Fig. 1B). The fasting blood glucose level in two months postpartum HIP females was similar to that measured at baseline (Fig. 1D), indicating that they were still in a pre-diabetic state. In fact, fasting blood glucose was similar in all HIP and WT groups throughout the study (see time 0 in Supplementary Fig. 2). Glucose tolerance did not change significantly over this time in either WT or HIP females from the control, non-pregnant group (Fig. 1B,C). Thus, heterozygous HIP females display the typical glucose intolerance of GDM. WT females were used a model of normal, uncomplicated pregnancy.

### Cardiac hypertrophy after a GDM-complicated pregnancy in rat females

**Figure 2:**
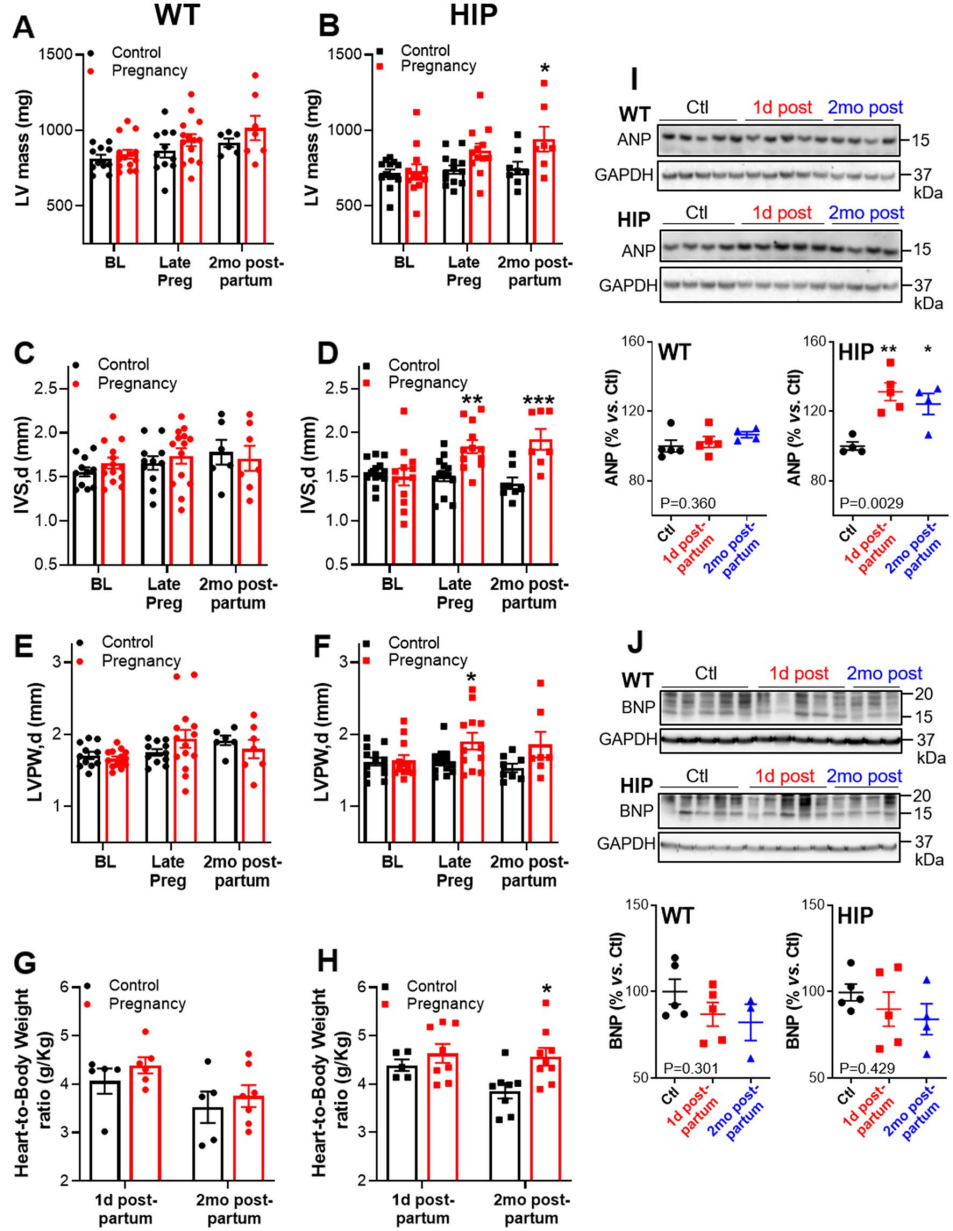
Cardiac hypertrophy in two months postpartum HIP females. (**A**–**F**) Left ventricular mass (**A,B**), diastolic thickness of the interventricular septum (**C,D**) and diastolic thickness of the left-ventricular posterior wall (**E,F**) in WT (**A,C,E**) and HIP (**B,D,F**) females from control and pregnancy groups at baseline (BL), late pregnancy and 2 months postpartum. (**G,H**) Heart weight-to-body weight ratio of WT (**G**) and HIP (**H**) females at one day and 2 months postpartum. (**I,J**) Expression of ANP (**I**) and BNP (**J**) in hearts from control (Ctl), one day postpartum and 2 months postpartum WT and HIP females. Top panels show representative examples while bottom panels show relative band intensities; the experiment was repeated 4 times. For BNP, the intensity analysis included the bands with molecular weights between 15 and 20 kDa. In panels (**A–H**), statistical significance was determined using Two-way ANOVA with the Bonferroni post-test to compare the pregnancy versus control groups at each timepoint. In panels (**I,J**), data for animals in control (not pregnant) groups sacrificed at the time when pregnancy groups were at 1 day and 2 months postpartum were pooled. For these panels, statistical analysis was performed using One-way ANOVA (*P* value is indicated on the graph) with Dunnett’s post-test for multiple comparisons.

Transthoracic echocardiography was used to monitor heart size and function in WT and HIP females from the control and pregnancy groups at baseline, late pregnancy and two months postpartum (Tables 1, 2, Fig. 2A–F, Supplemental Fig. 3). In HIP females, the left-ventricular mass (Fig. 2B), thickness of interventricular septum (Fig. 2D) and thickness of the left-ventricular posterior wall (Fig. 2F) were significantly increased in the pregnancy versus control group in late pregnancy and remained elevated two months after giving birth. In contrast, heart size was not significantly changed during pregnancy and postpartum periods in WT females from the pregnancy group compared to the WT control group (Fig. 2A,C,E).

**Table 1:** Echocardiography parameters in WT females from control and pregnancy groups.

WT females	Baseline		Late Pregnancy		Postpartum (2 mo)	
N	Control 11	Pregnancy 14	Control 11	Pregnancy 14	Control 6	Pregnancy 7
HR (bpm)	325 ± 8	332 ± 7	331 ± 14	349 ± 10	356 ± 17	331 ± 18
FS (%)	45.3 ± 1.7	46.4 ± 1.8	46.9 ± 2.1	54.0 ± 2.7	50.9 ± 2.3	47.8 ± 3.5
EF (%)	74.8 ± 2.0	75.9 ± 1.9	76.3 ± 2.3	82.6 ± 2.2	80.7 ± 2.3	76.9 ± 3.1
IVS,d (mm)	1.56 ± 0.05	1.65 ± 0.06	1.66 ± 0.08	1.74 ± 0.09	1.78 ± 0.14	1.71 ± 0.14
IVS,s (mm)	2.83 ± 0.07	2.93 ± 0.10	2.91 ± 0.10	3.41 ± 0.12*	3.38 ± 0.25	3.29 ± 0.24
LVID,d (mm)	7.23 ± 0.13	7.24 ± 0.10	7.18 ± 0.14	7.29 ± 0.22	7.21 ± 0.39	7.79 ± 0.26
LVID,s (mm)	3.96 ± 0.17	3.89 ± 0.16	3.82 ± 0.19	3.40 ± 0.27	3.66 ± 0.16	4.12 ± 0.33
LVPW,d (mm)	1.71 ± 0.05	1.66 ± 0.03	1.75 ± 0.05	1.94 ± 0.12	1.92 ± 0.07	1.80 ± 0.13
LVPW,s (mm)	2.77 ± 0.09	2.75 ± 0.08	2.89 ± 0.11	3.43 ± 0.23*	3.25 ± 0.10	3.09 ± 0.21
LV Mass (mg)	815 ± 25	843 ± 29	864 ± 44	936 ± 40	918 ± 28	1015 ± 81

Measurements were performed at baseline, during late pregnancy and two months after giving birth.

*HR* heart rate, *bpm* beats per minute, *FS* fractional shortening, *EF* ejection fraction, *IVS,d and IVS,s* thickness of interventricular septum in diastole and systole, respectively, *LVID,d* and *LVID,s* left-ventricular internal diameter in diastole and systole, respectively, *LVPW,d* and *LVPW,s* thickness of left-ventricular posterior wall in diastole and systole, respectively. *P < 0.05 vs. control group.

**Table 2:** Echocardiography parameters in HIP females from control and pregnancy groups.

HIP females	Baseline		Lategrant Pregnancy		Postpartum (2 mo)	
N	Control 13	Pregnancy 12	Control 13	Pregnancy 12	Control 8	Pregnancy 7
HR (bpm)	333 ± 7	333 ± 9	333 ± 13	357 ± 11	338 ± 13	365 ± 16
FS (%)	44.1 ± 1.1	43.3 ± 1.9	45.9 ± 2.8	48.0 ± 2.4	46.6 ± 1.9	46.6 ± 3.9
EF (%)	73.9 ± 1.2	72.6 ± 2.3	74.6 ± 3.5	77.0 ± 2.4	76.2 ± 2.1	75.5 ± 4.0
IVS,d (mm)	1.55 ± 0.04	1.50 ± 0.10	1.51 ± 0.06	1.84 ± 0.07**	1.43 ± 0.06	1.92 ± 0.12***
IVS,s (mm)	2.69 ± 0.05	2.64 ± 0.12	2.78 ± 0.10	3.08 ± 0.09	2.84 ± 0.11	3.41 ± 0.17**
LVID,d (mm)	6.81 ± 0.12	6.99 ± 0.20	7.25 ± 0.25	6.63 ± 0.23	7.45 ± 0.16	6.87 ± 0.45
LVID,s (mm)	3.81 ± 0.12	3.97 ± 0.18	3.96 ± 0.32	3.50 ± 0.22	3.97 ± 0.13	3.51 ± 0.50
LVPW,d (mm)	1.62 ± 0.06	1.64 ± 0.07	1.62 ± 0.05	1.91 ± 0.12*	1.54 ± 0.06	1.86 ± 0.18
LVPW,s (mm)	2.57 ± 0.08	2.54 ± 0.09	2.71 ± 0.07	3.11 ± 0.15	2.53 ± 0.07	2.97 ± 0.34
LV Mass (mg)	716 ± 25	728 ± 48	740 ± 28	866 ± 46	750 ± 42	941 ± 82*

Measurements were performed at baseline, during late pregnancy and two months after giving birth. * *P* < 0.05, ** *P* < 0.01, and *** *P* < 0.001 vs. control group.

**Figure 3:**
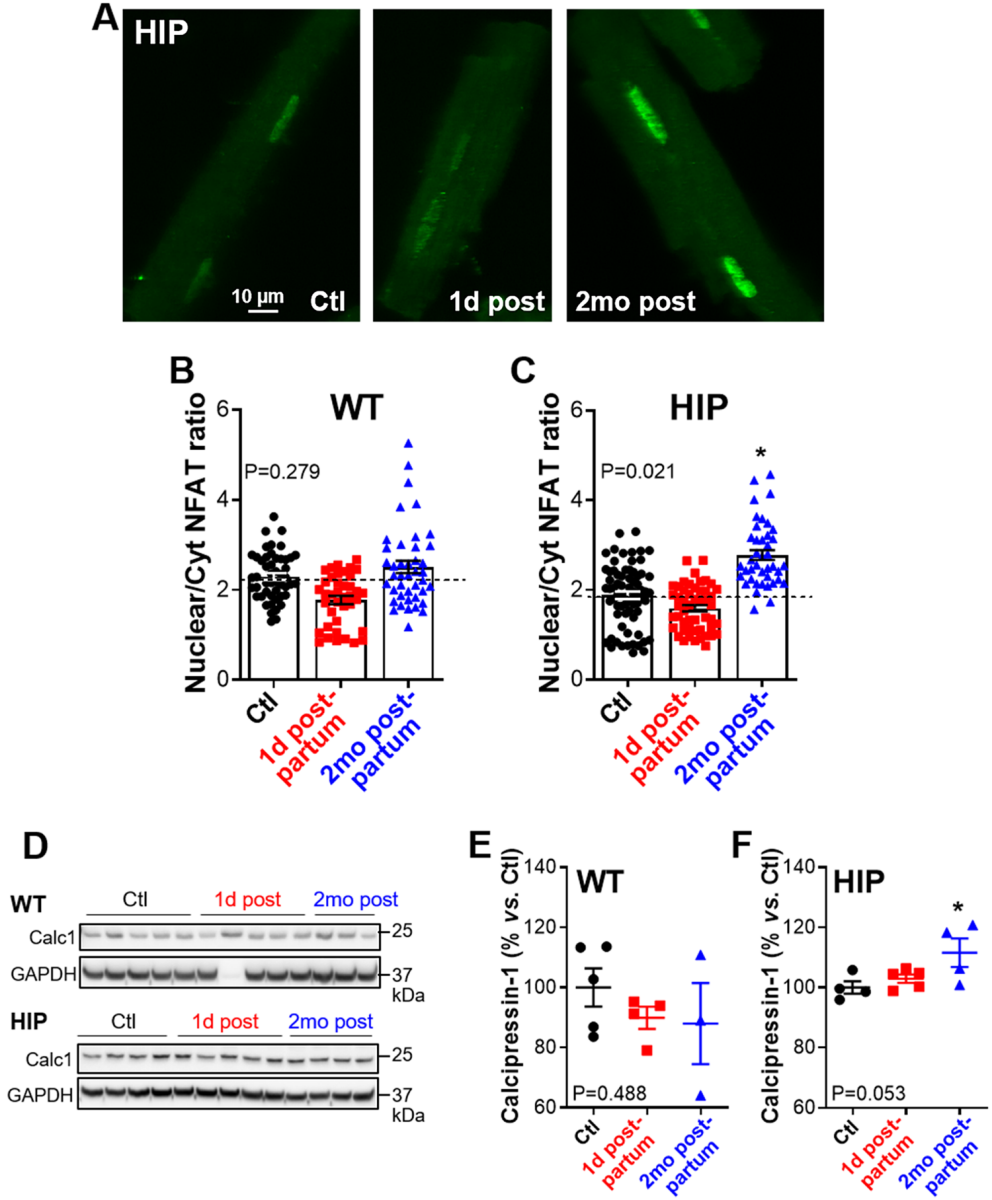
Nuclear versus cytosolic localization of NFATc4 in myocytes from HIP and WT females. (**A**) Examples of immunofluorescence images in myocytes from HIP females in the control (Ctl), 1 day postpartum (1d post) and 2 months postpartum (2mo post) groups. Cells were labeled with an anti-NFATc4 antibody. (**B,C**) Mean ratio of nuclear-to-cytosolic NFATc4 signal in cells from WT (**B**) and HIP (**C**) females in the control (WT: 4 females, 10–15 cells/rat; HIP: 6 females, 9–14 cells/rat), 1 day postpartum (WT: 3 females, 11–16 cells/rat; HIP: 5 females, 7–13 cells/rat) and 2 months postpartum (WT: 4 females, 10–11 cells/rat; HIP: 4 females, 9–12 cells/rat) groups. The number of cells per each rat is shown in Supplementary Table 1. (**D–F**) Expression of calcipressin-1 in hearts from control (Ctl), one day postpartum and two months postpartum WT and HIP females. Representative examples (**D**) and relative band intensities (**E,F**). Data for the sample loaded into Lane 7 of the WT gel were excluded from analysis. Data for animals in control groups sacrificed at the time when pregnancy groups were at 1 day and 2 months postpartum were pooled. Statistical analysis was performed with a mixed model using the Nested One-Way ANOVA analysis in Prism 8.1.2 (panels **B** and **C**; *P* values are shown on the graph) or One-way ANOVA (panels **E** and **F**; *P* values are on the graph) and Dunnett’s post-test for multiple comparisons.

In agreement with the echocardiography data, the heart weight-to-body weight ratio was elevated two months after giving birth in HIP females from the pregnancy group compared to HIP females from the control group, while no significant differences occurred in WT females (Fig. 2G,H). Moreover, the level of the hypertrophic marker ANP was increased in hearts from postpartum HIP females but not in postpartum WT females compared to their respective non-pregnant controls (Fig. 2I). The BNP level did not change with pregnancy in either HIP or WT females (Fig. 2J).

Together, these data indicate the presence of cardiac hypertrophy in the postpartum period following a GDM-complicated pregnancy (HIP rats) but not a normal pregnancy (WT rats).

### Enhanced activity of calcineurin/NFAT hypertrophy signaling in postpartum HIP rat females following a GDM-complicated pregnancy

Since the calcineurin/NFAT hypertrophy pathway is activated in both type-2 diabetes and early stages of normal pregnancies, we investigated whether enhanced calcineurin/NFAT signaling contributes to the cardiac hypertrophy following a GDM-complicated pregnancy. In this pathway, Ca^2+^/calmodulin-dependent activation of the phosphatase calcineurin leads to de-phosphorylation of NFAT, causing its translocation into the nucleus where it activates gene transcription^37^. Thus, the activity of calcineurin/ NFAT pathway was assessed from the nuclear *versus* cytosolic localization of NFATc4 as measured by immunofluorescence in isolated cardiac myocytes (Fig. 3A–C). There was a tendency towards lower nuclear-to-cytosolic NFATc4 ratio immediately (within 1 day) postpartum in hearts from both WT and HIP females compared to the respective control groups (Fig. 3A–C), in agreement with data indicating downregulation of this signaling pathway in late pregnancy^18,29^. Two months after giving birth, this pathway returned to its baseline activation level in WT females (Fig. 3B). However, the ratio of nuclear-to-cytosolic NFATc4 was significantly larger in myocytes from two months postpartum HIP females compared to control (Fig. 3A,C), indicating re-activation of this hypertrophy pathway following a GDM-complicated pregnancy.

To further evaluate the activity of calcineurin/NFAT pathway, we used immunoblot to measure the expression of calcipressin-1 (also known as RCAN1 or MCIP1), an endogenous calcineurin inhibitor whose expression is under the control of calcineurin/NFAT^38–40^. Calcipressin-1 expression was comparable in hearts from control, 1 day postpartum and two months postpartum WT females (Fig. 3D,E). In contrast, calcipressin expression was mildly but significantly elevated in hearts from two months postpartum HIP females (Fig. 3D,F).

**Figure 4:**
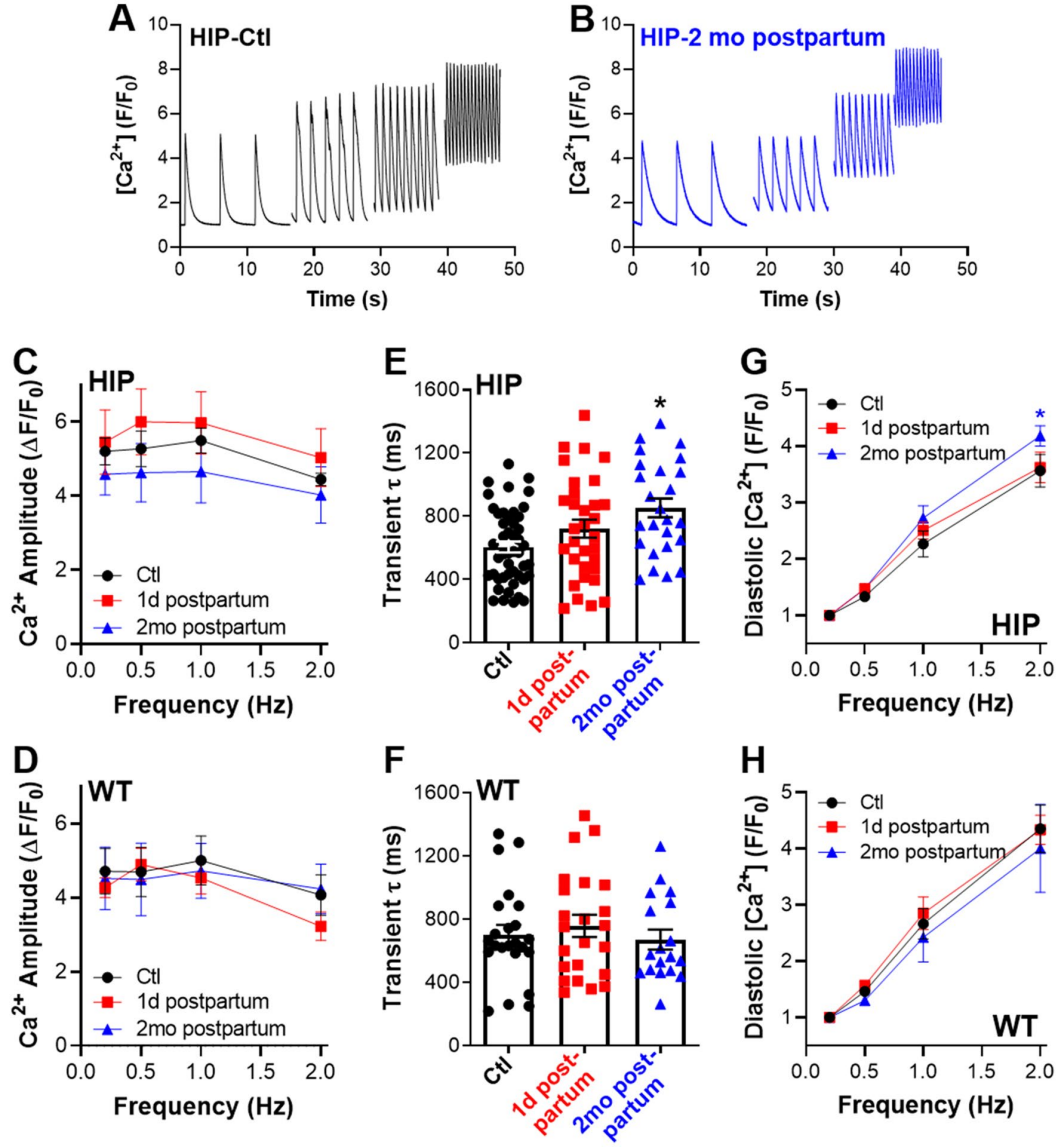
Ca^2+^ transients in myocytes from control (Ctl), 1 day and 2 months postpartum females. (**A**,**B**) Representative examples of Ca^2+^ transients stimulated at 0.2, 0.5, 1 and 2 Hz in myocytes from control, non-pregnant HIP females (**A**) and two months postpartum HIP females (**B**). (**C,D**) Ca^2+^ transient amplitude in myocytes from HIP (**C**) and WT (**D**) females. (**E,F**) Decay time of Ca^2+^ transients triggered by pacing at 0.5 Hz in myocytes from HIP (**E**) and WT (**F**) females. (**G,H**) Diastolic Ca^2+^ as a function of stimulation frequency in myocytes from HIP (**G**) and WT (**H**) females. Measurements were done in females from control (WT: 6 females, 3-4cells/rat; HIP: 9 females, 3–7 cells/rat), 1 day postpartum (WT: 6 rats, 3–6 cells/rat; HIP: 8 rats, 2–6 cells/rat) and 2 months postpartum (WT: 3 rats, 6 cells/rat; HIP: 5 rats, 3–7 cells/rat) groups. The number of cells per each rat is shown in Supplementary Table 2. Data for animals in control groups sacrificed at the time when pregnancy groups were at 1 day and 2 months postpartum were pooled. Statistical analysis was performed with Two-way ANOVA for panels C, D, G and H, and with a mixed model using the Nested One-Way ANOVA analysis in Prism 8.1.2 for panels E and F. Dunnett’s post-test was used to account for multiple comparisons.

Since calcineurin is activated by an increase in cytosolic Ca^2+^ concentration, we measured Ca^2+^ transients triggered by field stimulation at various frequencies between 0.2 and 2 Hz in myocytes from control, one day postpartum and two months postpartum WT and HIP females (Fig. 4A,B). Ca^2+^ transient amplitude was not significantly affected by the pregnancy and postpartum stages in either HIP or WT females (Fig. 4C,D). However, Ca^2+^ declined more slowly in myocytes from 2 months postpartum versus control, not-pregnant HIP females (Fig. 4E), which resulted in a more pronounced rise in diastolic Ca^2+^ levels upon an increase in the stimulation frequency (Fig. 4G). In contrast, no such differences occurred in myocytes from postpartum and control WT females (Fig. 4F,H).

### Activation of CaMKII/HDAC hypertrophy signaling in pregnant rat females

**Figure 5:**
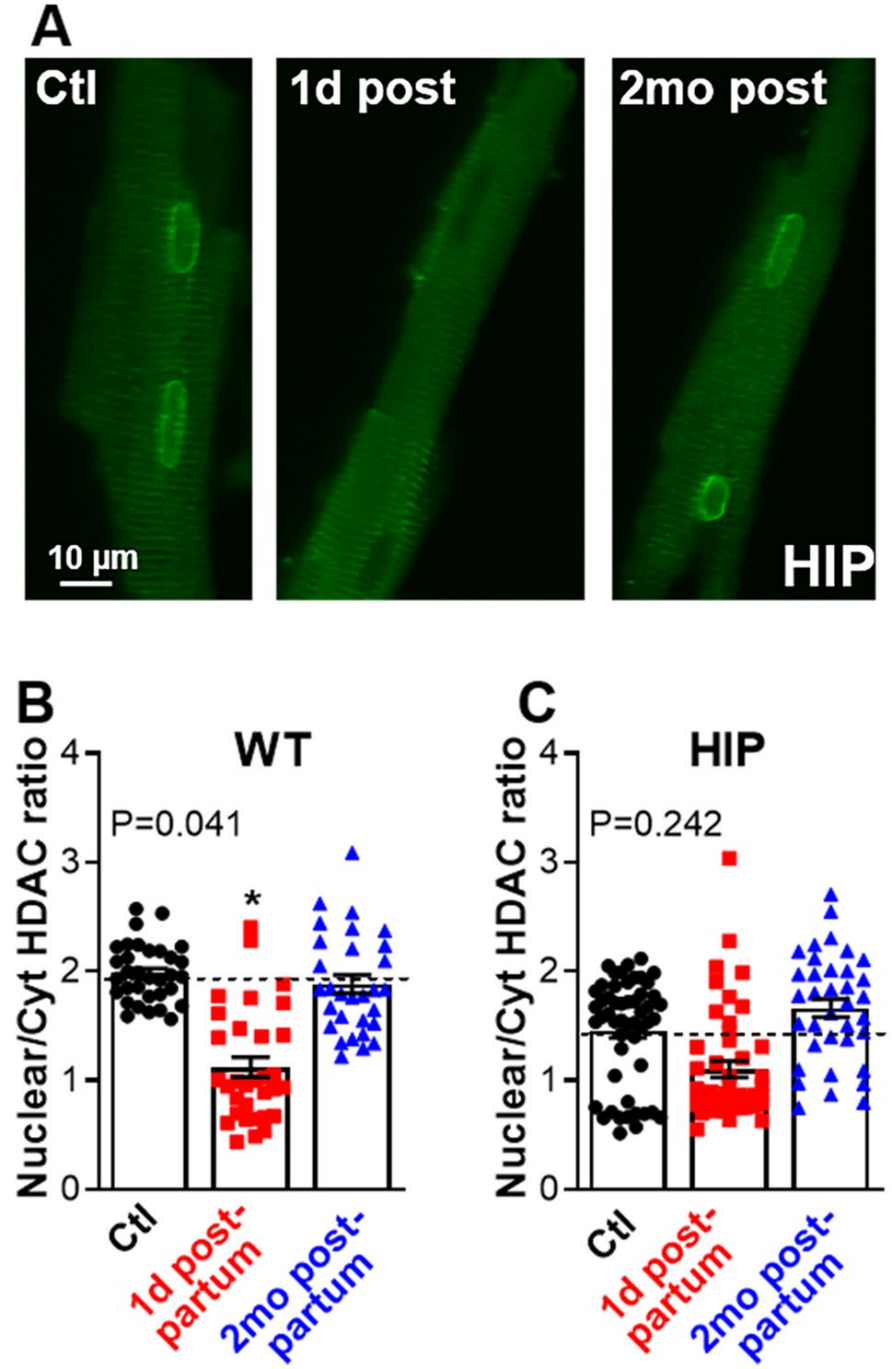
Nuclear versus cytosolic localization of HDAC4 in myocytes from HIP and WT females. (**A**) Examples of immunofluorescence images in myocytes from HIP females in the control (Ctl), 1 day postpartum (1d post) and 2 months postpartum (2mo post) groups. Cells were labeled with an anti-HDAC antibody. (**B,C**) Mean ratio of nuclear to cytosolic signal in cells from WT (**B**) and HIP (**C**) females in the control (WT: 3 females, 10–12 cells/rat; HIP: 5 females, 9–12 cells/rat), 1 day postpartum (WT: 3 females, 10–12 cells/rat; HIP: 4 females, 9–12 cells/rat) and 2 months postpartum (WT: 3 females, 10–11 cells/rat; HIP: 4 females, 6–10 cells/rat) groups. The number of cells per each rat is shown in Supplementary Table 3. Data for animals in control groups sacrificed at the time when pregnancy groups were at 1 day and 2 months postpartum were pooled. Statistical analysis was performed with a mixed model using the Nested One-Way ANOVA analysis in Prism 8.1.2 and Dunnett’s post-test for multiple comparisons.

CaMKII/HDAC signaling is another Ca^2+^-activated hypertrophy pathway in which, upon phosphorylation by CaMKII, HDAC moves out of the nucleus, which facilitates gene expression^41^. Using immunofluorescent staining of isolated myocytes, we found that HDAC4 is exported from the nucleus in hearts from WT and HIP females immediately after giving birth (Fig. 5), which suggests that the CaMKII/HDAC pathway is activated in both normal pregnancy and GDM. However, this pathway returned to its baseline activation level by two months postpartum in both WT and HIP females (Fig. 5).

## Discussion

Several population-based retrospective studies in large human cohorts established a strong association between GDM and the development of cardiovascular disease later in life^7–13^, including cardiac remodeling and dysfunction^12,14,15^. Using female rats that express the human variant of the pancreatic hormone amylin specifically in the β-cells (HIP rats) as a GDM model and their WT littermates as controls, we found here that pathological cardiac hypertrophy is also present two months post-delivery in female rats with GDM-complicated pregnancies but not in females with normal pregnancies (Fig. 2). Human studies cannot discriminate between GDM as a cause of the ulterior cardiovascular disease or a condition that develops in, and thus identifies, women that already are at higher risk. Since we compared postpartum HIP females with HIP females that did not go through pregnancy, our data suggest that GDM triggers, or at minimum accelerates, the postpartum development of pathological cardiac remodeling/ hypertrophy.

During a normal pregnancy, the heart generally undergoes physiological, mild eccentric hypertrophy, characterized by a proportional increase in chamber size and wall thickness^18,42–44^, as expected for volume overload-induced heart growth. However, one study reported increased thickness of the septal and posterior walls with no change in diastolic diameter in pregnant women^16^, while an investigation in pregnant mice found hypertrophy characterized by decreased left-ventricular wall thickness and larger chamber size^19^. Somewhat surprisingly, pregnant WT females did not show overt cardiac remodeling or hypertrophy in our study. The reasons for this are unclear but might reflect reduced ability of the heart to undergo physiological hypertrophy due to a more advanced age (~ 5.5 months of age). This is in line with studies reporting lack of exercise-induced hypertrophy in aged animals^45,46^. In contrast, our data suggest that GDM is associated with concentric remodeling/hypertrophy, both at term and postpartum, as HIP females showed increased diastolic thickness of both interventricular septum (Fig. 2D) and posterior wall (Fig. 2F) with no significant change in left-ventricular diameter (Table 2) compared to HIP females that did not experience pregnancy. This is consistent with human data showing larger left-ventricular wall index at term in females with GDM compared to females with normal pregnancies^14,15^.

Cardiac hypertrophy observed two months postpartum in female rats with GDM was associated with activation of calcineurin/NFAT signaling, as evidenced by the nuclear translocation of NFATc4 (Fig. 3A,C) and increased expression of calcipressin-1, a protein whose expression is often used as an indicator of NFAT activation by calcineurin (Fig. 3F). Calcineurin is activated by an increase in cytosolic Ca^2+^ through binding of Ca^2+^/calmodulin. We found that Ca^2+^ transient decay is slower and consequently diastolic Ca^2+^ is higher in myocytes from two months postpartum *versus* control, not-pregnant HIP females (Fig. 4E). Thus, following each heartbeat Ca^2+^ stays in the cytosol longer, which may underlie calcineurin activation.

The calcineurin/NFAT pathway is usually involved in pathological hypertrophy, caused for example by pressure overload. However, this pathway is also activated in the early stage of normal pregnancies and is required for the pregnancy-induced heart growth^18,29^. Activation of calcineurin in early pregnancy was attributed to higher progesterone levels^29^, but the underlying mechanisms are not fully elucidated. We previously reported that calcineurin/NFAT signaling is activated in pre-diabetic, insulin-resistant male HIP rats through a mechanism related to cardiac deposition of aggregated amylin^28^, a hormone that is hypersecreted along with insulin by the pancreatic β-cells in pre-diabetes. HIP rats express the human isoform of amylin in the pancreas. In contrast to rodent amylin, the human variant is amyloidogenic^32^, which favors amylin aggregation and deposition in the pancreas and peripheral organs, including the heart^28,47,48^. In pre-diabetic HIP rats, amylin deposition leads to an increase in the sarcolemmal permeability to Ca^2+^ and thus raises cytosolic Ca^2+^ level, which activates calcineurin^28^. Females with GDM exhibit glucose intolerance, which is usually associated with an increase in insulin and amylin secretion. Thus, the cardiac stress caused by higher levels of circulating amyloid-forming amylin may also contribute to calcineurin/NFAT activation in females with GDM.

CaMKII/HDAC signaling is another Ca^2+^-dependent pathway that leads to expression of pro-hypertrophic genes. In contrast to calcineurin signaling, this pathway was active immediately after giving birth and returned to baseline by two months postpartum in both HIP and WT females (Fig. 5). This result suggests that the CaMKII/HDAC pathway may contribute to cardiac hypertrophy during pregnancy but not to the GDM-induced programing of heart growth later in life.

One limitation of the current study is that similar experiments cannot be performed in humans, which leaves open the question whether the mechanism identified here does account for the postpartum cardiac hypertrophy in women with prior GDM. Partial mechanistic validation could come however from studies in additional animal models. Moreover, the mechanism needs to be further validated by experiments where the calcineurin/NFAT pathway is inhibited during pregnancy.

In summary, we found that GDM results in concentric cardiac hypertrophy, likely due to activation of calcineurin/NFAT pathway, two months after giving birth in female rats. The comparison of postpartum HIP females with non-pregnant HIP females suggests a causative role for GDM in the postpartum development of pathological remodeling of the heart. Understanding the mechanisms through which GDM predisposes the mother to heart dysfunction, coupled with early detection of GDM, will allow clinicians to design effective lifestyle and/or pharmacological interventions during the pregnancy and postpartum periods to reduce the risk.

## Methods

The study is reported in accordance with ARRIVE guidelines.

### Experimental animals

All animal experiments were performed in accordance with the NIH Guide for the Care and Use of Laboratory Animals and were approved by the Institutional Animal Care and Use Committee at the University of Kentucky. N = 53 obese Sprague–Dawley female rats that are heterozygous for expression of the human isoform of the pancreatic hormone amylin specifically in the β-cells (HIP rats^28,30,31^) and N = 40 wild-type (WT) littermates were used in this study. HIP (25.4 ± 0.3 weeks of age) and WT (24.6 ± 0.5 weeks of age) females were randomly assigned to pregnancy and control study groups. Females in the pregnancy groups were paired for breeding with Sprague–Dawley males. N = 6 females assigned to pregnancy groups did not become pregnant and were excluded from the study. Females in both pregnancy and control groups were further randomized for euthanasia and heart collection at one day or two months postpartum. Rats had ad libitum access to food and water. At the end of the study, rats were anesthetized with 3–5% isoflurane (100% O_2_) and deep anesthesia was verified by lack of reflex upon toe pinch. Rats were then euthanized by exsanguination following excision of the heart.

### Glucose tolerance test

Standard glucose tolerance tests (GTT) were performed at baseline and when the pregnancy groups were at 18–20 days of pregnancy, 21 days postpartum and 2 months postpartum. Rats were fasted for 8 h, injected with glucose (2 g/kg of body weight, IP) and blood glucose was monitored every 15–30 min for two hours using a glucometer (OneTouch Ultra).

### Echocardiography

Transthoracic echocardiography was performed under anesthesia (0.5–3% isoflurane) at baseline and when the pregnancy groups were at 18–20 days of pregnancy and two months postpartum using a Vevo 2100 high-frequency ultrasound (VisualSonics, Toronto, ON, Canada). Two-dimensional imaging was used to identify the short-axis position. Five consecutive M-mode images in the short-axis view were then used for analysis of chamber size and heart function.

### Immunoblot

Hearts were homogenized in homogenization buffer containing 150 mM NaCl, 50 mM Tris–HCl, 50 mM NaF, 2% Triton X-100, 0.1% SDS, supplemented with phosphatase and proteases inhibitor cocktail set III (Calbiochem). Homogenates were then loaded onto polyacrylamide gels for SDS-PAGE electrophoresis, transferred to PVDF membranes, blocked with 5% milk and probed with primary antibodies against atrial natriuretic factor (ANP; Millipore AB2232, 1:1000), brain natriuretic peptide (BNP; Abcam ab239510, 1:1000), and calcipressin-1 (ThermoFisher 14869-1-AP, 1:1000). Equal protein loading was verified by re-probing for GAPDH (Abcam ab8245). Bands were detected by chemiluminescent signals using the enhanced chemiluminescence method (SuperSignal West Dura Extended Duration Substrate, ThermoScientific, USA) and visualized with a G:BOX gel imaging system (SynGene, Cambridge, United Kingdom). Band intensity was measured using ImageJ software (NIH, Bethesda, USA). For each gel, the signal intensity was averaged over the control samples. Then, the signal intensity in all lanes was normalized to this average. This procedure was repeated on all technical replicates and the normalized signal intensity was averaged for each sample, followed by averaging over experimental groups.

### Ventricular myocyte isolation

Rats were anesthetized with 3–5% isoflurane (100% O_2_) and hearts were excised by cutting the aorta, mounted on a gravity-driven Langendorff perfusion system and perfused with a nominally Ca^2+^ free medium containing 11.2 g/L Minimum Essential Medium (MEM; Sigma M0518), 40 Units/L human insulin, 10 mL/L Penicillin–Streptomycin, 4.8 mM NaHCO_3,_ 2 mM sodium pyruvate, 10 mM Na-HEPES, 10 mM HEPES and 3.5 μL/mL heparin (pH = 7.4) for 5 min to clear the blood. The heart was then perfused with a similar medium except that heparin was excluded and 0.1 mg/mL Liberase TH (Roche-Sigma), 30 μM CaCl_2_ and 8 mM taurine were added. When the heart became flaccid (~ 20 min), the tissue was cut into small pieces, dispersed, and filtered and the myocyte suspension was rinsed several times. Myocytes were kept at low Ca^2+^ (30 μM) until ready to use.

### Immunofluorescence

Freshly isolated myocytes were plated onto laminin-coated 8-well glass coverslips and fixed with 4% paraformaldehyde. Cells were then permeabilized with 50 µg/mL saponin (15 min), blocked with 10% goat serum and 10% BSA for one hour and incubated overnight at 4 °C with primary antibodies against NFATc4 (Santa Cruz sc-13036; 1:100 dilution) or HDAC4 (Santa Cruz sc-11418; 1:100 dilution). Myocytes were then washed (6x) with PBS containing 1% BSA and incubated with Alexa Fluor 488 conjugated anti-rabbit secondary antibody (ThermoFisher A11034; 1:50 dilution) for 2 h at room temperature. Cells were washed again in PBS and imaged with a laser scanning confocal microscope. Signal intensities were analyzed in Image J.

### Ca^2+^ transient measurements

Freshly isolated myocytes were loaded with Fluo-4 AM (10 μM for 20 min) and paced at 0.2, 0.5, 1 and 2 Hz through platinum electrodes in a standard Tyrode’s solution containing (in mM): 140 NaCl, 4 KCl, 1 MgCl_2_, 10 glucose, 5 HEPES, and 1 CaCl_2_ (pH = 7.4). Cells were paced for > 2 min at each frequency to allow Ca^2+^ transients and contractions to reach steady-state. The transients were the recorded for 10–15 s using a Nikon Eclipse Ti microscope equipped with a Lambda DG-4 illumination system (Sutter Instrument, Novato, CA) and an Andor iXon CCD camera (Andor Technology, Belfast, UK). All experiments were done at room temperature (23–25 °C) and results are expressed as F/F_0_, where F_0_ is the fluorescence intensity when the cell is at rest.

### Statistical analysis

Data are presented as mean ± SEM. For data in Figs. 1, 2, and 3D, statistical analysis of differences between groups was performed in Prism 8.1.2 using the student’s t-test, One-way ANOVA with Dunnett’s multiple comparisons test or Two-way ANOVA with Bonferroni post-test, as appropriate. Single-cell experiments in Figs. 3, 4 and 5 were analyzed with a mixed model using the Nested One-Way ANOVA analysis with Dunnett’s multiple comparisons post-test in Prism 8.1.2 software. Differences were considered statistically significant when *P* < 0.05. *P < 0.05, **P < 0.01, ***P < 0.001, ****P < 0.0001.

## Supplementary Information

Supplementary Information.

## References

[1] Johns EC, Denison FC, Norman JE, Reynolds RM (2018). Gestational diabetes mellitus: Mechanisms, treatment and complications. Trends Endocrinol. Metab..

[2] Sacks DA (2012). HAPO Study Cooperative Research Group. Frequency of gestational diabetes mellitus at collaborating centers based on IADPSG consensus panel-recommended criteria: The Hyperglycemia and Adverse Pregnancy Outcome (HAPO) Study. Diabetes Care.

[3] DeSisto CL, Kim SY, Sharma AJ (2014). Prevalence estimates of gestational diabetes mellitus in the United States, Pregnancy Risk Assessment Monitoring System (PRAMS), 2007–2010. Prev. Chronic Dis..

[4] Chu SY (2007). Maternal obesity and risk of gestational diabetes mellitus. Diabetes Care.

[5] Kim C, Newton KM, Knopp RH (2002). Gestational diabetes and the incidence of type 2 diabetes: A systematic review. Diabetes Care.

[6] Bellamy L, Casas JP, Hingorani AD, Williams D (2009). Type 2 diabetes mellitus after gestational diabetes: A systematic review and meta-analysis. Lancet.

[7] Daly B (2018). Increased risk of ischemic heart disease, hypertension, and type 2 diabetes in women with previous gestational diabetes mellitus, a target group in general practice for preventive interventions: A population-based cohort study. PLoS Med..

[8] Goueslard K (2016). Early cardiovascular events in women with a history of gestational diabetes mellitus. Cardiovasc. Diabetol..

[9] Shah BR, Retnakaran R, Booth GL (2008). Increased risk of cardiovascular disease in young women following gestational diabetes mellitus. Diabetes Care.

[10] Kessous R, Shoham-Vardi I, Pariente G, Sherf M, Sheiner E (2013). An association between gestational diabetes mellitus and long-term maternal cardiovascular morbidity. Heart.

[11] McKenzie-Sampson S, Paradis G, Healy-Profitós J, St-Pierre F, Auger N (2018). Gestational diabetes and risk of cardiovascular disease up to 25 years after pregnancy: A retrospective cohort study. Acta Diabetol..

[12] Appiah D (2016). Association of gestational diabetes mellitus with left ventricular structure and function: The CARDIA study. Diabetes Care.

[13] Kramer CK, Campbell S, Retnakaran R (2019). Gestational diabetes and the risk of cardiovascular disease in women: A systematic review and meta-analysis. Diabetologia.

[14] Buddeberg BS (2020). The impact of gestational diabetes on maternal cardiac adaptation to pregnancy. Ultrasound Obstet. Gynecol..

[15] Oliveira AP (2015). Assessment of structural cardiac abnormalities and diastolic function in women with gestational diabetes mellitus. Diab. Vasc. Dis. Res..

[16] Schannwell CM (2002). Left ventricular hypertrophy and diastolic dysfunction in healthy pregnant women. Cardiology.

[17] Savu O (2012). Morphological and functional adaptation of the maternal heart during pregnancy. Circ. Cardiovasc. Imaging.

[18] Chung E, Leinwand LA (2014). Pregnancy as a cardiac stress model. Cardiovasc. Res..

[19] Eghbali M (2005). Molecular and functional signature of heart hypertrophy during pregnancy. Circ. Res..

[20] Goldstein J, Sites CK, Toth MJ (2004). Progesterone stimulates cardiac muscle protein synthesis via receptor-dependent pathway. Fertil. Steril..

[21] Chung E, Yeung F, Leinwand LA (2012). Akt and mapk signaling mediate pregnancy-induced cardiac adaptation. J. Appl. Physiol..

[22] Devereux RB (2000). Impact of diabetes on cardiac structure and function: The strong heart study. Circulation.

[23] Poornima IG, Parikh P, Shannon RP (2006). Diabetic cardiomyopathy: The search for a unifying hypothesis. Circ. Res..

[24] Szwejkowski BR (2013). Allopurinol reduces left ventricular mass in patients with type 2 diabetes and left ventricular hypertrophy. J. Am. Coll. Cardiol..

[25] Adeghate E, Singh J (2014). Structural changes in the myocardium during diabetes-induced cardiomyopathy. Heart Fail. Rev..

[26] Seferovic JP (2018). Increased left ventricular mass index is present in patients with type 2 diabetes without ischemic heart disease. Sci. Rep..

[27] Rospleszcz S (2018). Association of glycemic status and segmental left ventricular wall thickness in subjects without prior cardiovascular disease: A cross-sectional study. BMC Cardiovasc. Disord..

[28] Despa S (2012). Hyperamylinemia contributes to heart dysfunction in obesity and diabetes, a study in humans and rats. Circ. Res..

[29] Chung E, Yeung F, Leinwand LA (2013). Calcineurin activity is required for cardiac remodeling in pregnancy. Cardiovasc. Res..

[30] Butler AE (2004). Diabetes due to a progressive defect in beta-cell mass in rats transgenic for human islet amyloid polypeptide (HIP Rat): A new model for type 2 diabetes. Diabetes.

[31] Matveyenko AV, Butler PC (2006). β-cell deficit due to increased apoptosis in the human islet amyloid polypeptide transgenic (HIP) rat recapitulates the metabolic defects present in type-2 diabetes. Diabetes.

[32] Westermark P, Andersson A, Westermark GT (2011). Islet amyloid polypeptide, islet amyloid, and diabetes mellitus. Physiol. Rev..

[33] Lutz TA (2012). Control of energy homeostasis by amylin. Cell Mol. Life Sci..

[34] Baldo BA, Kelley AE (2001). Amylin infusion into rat nucleus accumbens potently depresses motor activity and ingestive behavior. Am. J. Physiol. Regul. Integr. Comp. Physiol..

[35] Kreutter DK (1993). Amylin and CGRP induce insulin resistance via a receptor distinct from cAMP-coupled CGRP receptor. Am. J. Physiol..

[36] Castle AL, Kuo CH, Ivy JL (1998). Amylin influences insulin-stimulated glucose metabolism by two independent mechanisms. Am. J. Physiol.

[37] Hill JA, Olson EN (2008). Cardiac plasticity. N. Engl. J. Med..

[38] Yang J (2000). Independent signals control expression of the calcineurin inhibitory proteins MCIP1 and MCIP2 in striated muscles. Circ. Res..

[39] Camacho Londoño JE (2015). A background Ca^2+^ entry pathway mediated by TRPC1/TRPC4 is critical for development of pathological cardiac remodelling. Eur. Heart J..

[40] Parraa V, Rothermel BA (2017). Calcineurin signaling in the heart: The importance of time and place. J. Mol. Cell. Cardiol..

[41] Bers DM (2008). Calcium cycling and signaling in cardiac myocytes. Annu. Rev. Physiol..

[42] Melchiorre K, Sharma R, Khalil A, Thilaganathan B (2016). Maternal cardiovascular function in normal pregnancy: Evidence of maladaptation to chronic volume overload. Hypertension.

[43] Cong J (2015). Structural and functional changes in maternal left ventricle during pregnancy: A three-dimensional speckle-tracking echocardiography study. Cardiovasc. Ultrasound.

[44] Umar S (2012). Cardiac structural and hemodynamic changes associated with physiological heart hypertrophy of pregnancy are reversed postpartum. J. Appl. Physiol..

[45] Roh J, Rhee J, Chaudhari V, Rosenzweig A (2016). The role of exercise in cardiac aging: From physiology to molecular mechanisms. Circ. Res..

[46] Roh JD (2020). Exercise training reverses cardiac aging plenotypes associated with heart failure with preserved ejection fraction in male mice. Aging Cell.

[47] Despa S (2014). Cardioprotection by controlling hyperamylinemia in a "humanized" diabetic rat model. J. Am. Heart Assoc..

[48] Liu M (2016). Hyperamylinemia increases IL-1β synthesis in the heart via peroxidative sarcolemmal injury. Diabetes.

