# Supplementary material for: Gestational diabetes triggers postpartum cardiac hypertrophy via activation of calcineurin/NFAT signaling
Source: Sci Rep. 2021 Oct 22;11:20926. doi: 10.1038/s41598-021-00422-3 (PMC8536766; doi:10.1038/s41598-021-00422-3)
Supplement: Supplementary file 1 — Supplementary Information. [file 41598_2021_422_MOESM1_ESM.doc]

**Gestational diabetes triggers postpartum cardiac hypertrophy via activation of calcineurin/NFAT signaling**

Nirmal Verma, Sarah Srodulski, Sathya Velmurugan, Amanda Hoskins, Vivek K Pandey, Florin Despa, Sanda Despa

**Supplementary Material**

**Supplementary Methods**

**Measurement of plasma amylin level**

Plasma amylin level was measured using an ELISA assay kit (EZHA-52K, Millipore, MA) as per manufacturer’s protocol. A calibration curve was constructed by fitting the data in solutions with known amylin concentration (in the 1.3-175 pM range) with a Four Parameter Logistic Curve.

**Supplementary Figures**





**Supplementary Figure 1.** (A) Plasma amylin level at baseline in a subset of WT and HIP females used in the study. (B) Body weight at baseline in WT and HIP females.

**Supplementary Figure 2.** Time course of blood glucose level in glucose tolerance tests performed in HIP and WT females in the pregnancy (Preg; N=17 HIP and 14 WT in total) and control (Ctl; N=13 HIP and 11 WT in total) groups. Measurements were done in late (19-20 days) pregnancy, at the time of weaning the pups and two months after giving birth.


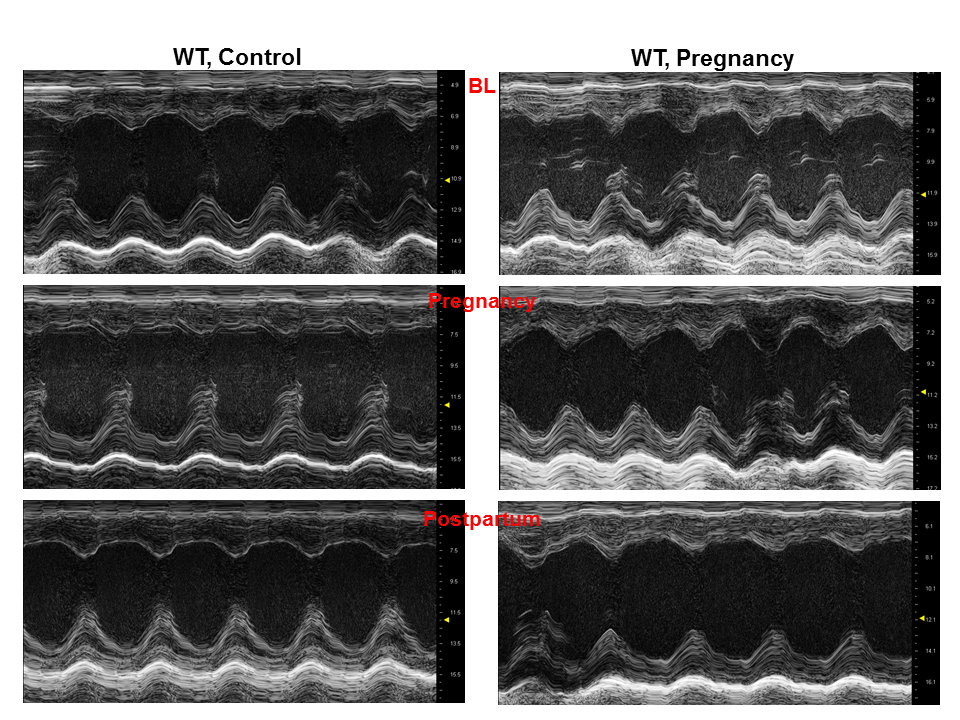

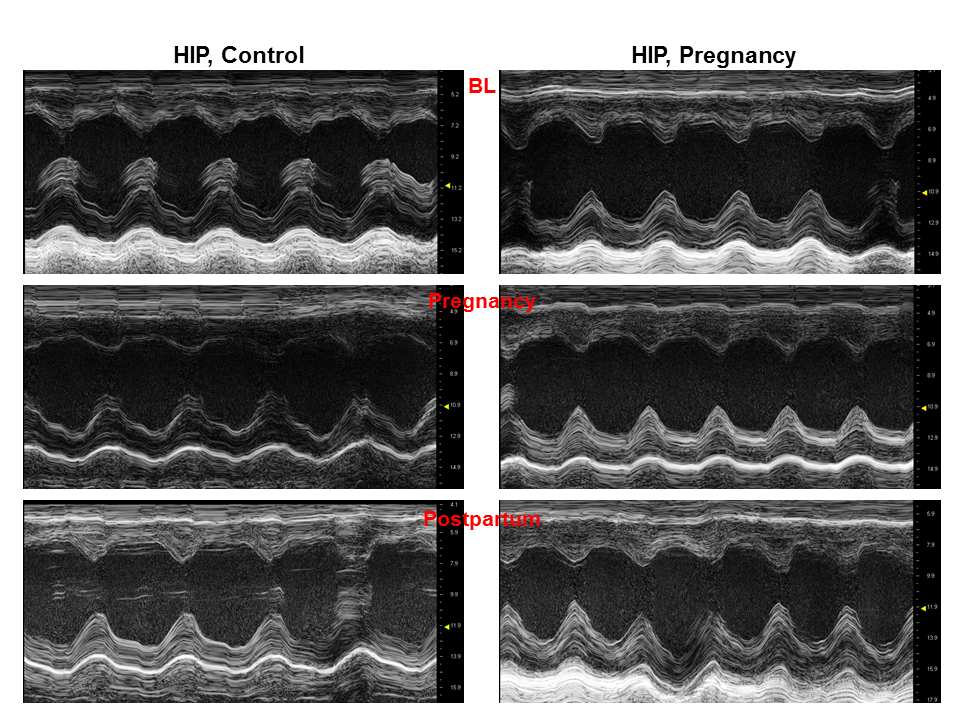
**Supplementary Figure 3.** Echocardiography recordings (M-mode) from HIP and WT females in the pregnancy and control groups. Measurements were performed at baseline (BL), late pregnancy (Pregnancy) and two months after giving birth (Postpartum).

**Supplementary Tables**

**Supplementary Table 1.** Number of cells from each rat used for experiments in Figure 3B-C.

|  | **WT females** | | | **HIP females** | | |
| --- | --- | --- | --- | --- | --- | --- |
|  | Control | 1 day postpartum | 2 months postpartum | Control | 1 day postpartum | 2 months postpartum |
| Rat #1 | 12 | 12 | 10 | 12 | 10 | 10 |
| Rat #2 | 12 | 11 | 10 | 9 | 11 | 10 |
| Rat #3 | 10 | 16 | 11 | 10 | 9 | 9 |
| Rat #4 | 15 |  | 11 | 14 | 13 | 12 |
| Rat #5 |  |  |  | 14 | 7 |  |
| Rat #6 |  |  |  | 11 |  |  |

**Supplementary Table 2.** Number of cells from each rat used for experiments in Figure 4.

|  | **WT females** | | | **HIP females** | | |
| --- | --- | --- | --- | --- | --- | --- |
|  | Control | 1 day postpartum | 2 months postpartum | Control | 1 day postpartum | 2 months postpartum |
| Rat #1 | 4 | 3 | 6 | 6 | 3 | 3 |
| Rat #2 | 4 | 6 | 6 | 3 | 2 | 5 |
| Rat #3 | 3 | 3 | 6 | 5 | 4 | 7 |
| Rat #4 | 4 | 4 |  | 5 | 5 | 6 |
| Rat #5 | 4 | 5 |  | 6 | 3 | 5 |
| Rat #6 | 4 | 5 |  | 4 | 5 |  |
| Rat #7 |  |  |  | 7 | 6 |  |
| Rat #8 |  |  |  | 7 | 4 |  |
| Rat #9 |  |  |  | 6 |  |  |

**Supplementary Table 3.** Number of cells from each rat used for experiments in Figure 5.

|  | **WT females** | | | **HIP females** | | |
| --- | --- | --- | --- | --- | --- | --- |
|  | Control | 1 day postpartum | 2 months postpartum | Control | 1 day postpartum | 2 months postpartum |
| Rat #1 | 10 | 10 | 10 | 10 | 9 | 10 |
| Rat #2 | 11 | 10 | 10 | 9 | 10 | 10 |
| Rat #3 | 12 | 12 | 11 | 9 | 12 | 10 |
| Rat #4 |  |  |  | 12 | 9 | 6 |
| Rat #5 |  |  |  | 12 |  |  |
